# Distribution of Immunohistochemical Subtypes of Breast Cancer in Nigeria: A Systematic Review

**DOI:** 10.7759/cureus.91806

**Published:** 2025-09-07

**Authors:** Christian O Igibah, Daniel Asogun, Osarenoma Mathilda Omonfuegbe, Mahmud A Mahmud, Atohengbe Aluede, Mitchel A Shula, Uche Smith, Julian Ojebo, Esteem Tagar, Oluwaseyifunmi Onabolu

**Affiliations:** 1 Research, Cancer Advocacy Nigeria, Irrua, NGA; 2 General Practice, Stockport NHS Foundation Trust, Stockport, GBR; 3 Urology, Sheffield Teaching Hospitals NHS Foundation Trust, Sheffield, GBR; 4 Medicine and Surgery, Ambrose Alli University, Ekpoma, NGA; 5 Medical Microbiology, University Hospitals Dorset NHS Foundation Trust, Poole, GBR; 6 Internal Medicne, Baptist Health-UAMS (University of Arkansas for Medical Sciences), Little Rock, USA; 7 Anaesthesiology, Garki Hospital, Abuja, NGA; 8 Surgery/General Surgery, Ambrose Alli University, Ekpoma, NGA; 9 Surgery/General Surgery, Irrua Specialist Teaching Hospital, Irrua, NGA

**Keywords:** breast cancer pathology, breast cancer research, epidemiology, immunohistochemistry and biopsy, nigeria

## Abstract

Breast cancer remains the most common cancer seen in women worldwide. Great strides have been made in its management even in low- and medium-income countries such as Nigeria. Immunohistochemistry is essential in planning treatment, with the increasing availability of hormonal and other targeted therapies. This systematic review aims to review the immunohistochemical subtypes of breast cancer seen in Nigeria. A literature search was done on PubMed, Google Scholar, and African Journal Online databases for publications on the immunohistochemical subtypes of breast cancer in Nigeria using the Preferred Reporting Items for Systematic Reviews and Meta-Analysis (PRISMA) guidelines. Articles were selected on the basis of their relevance to immunohistochemistry of breast cancer in Nigeria. A total of 17 studies published between 2005 and 2024, comprising 3,017 patients, met the inclusion criteria. Geographically, most were from the South West region (n=8), followed by South South (n=4), North Central (n=3), and one each from the South East and North West. Sixteen studies, with a total of 2,846 subjects, reported hormone receptor status. Estrogen receptor-positive (ER+) rates ranged from 10.9% to 65.1%, progesterone receptor-positive (PR+) rates from 12.5% to 70.7%, human epidermal growth factor receptor 2-positive (HER2+) rates from 5.2% to 62.5%, and triple-negative breast cancer* (*TNBC) rates from 12.2% to 79.3%. Ten studies (n = 2,220) reported molecular subtypes, with luminal A subtype ranging from 4.7% to 61.9%, luminal B from 2.1% to 22.8%, and Basal-like cancers ranging from 12.5% to 40.7%. Regional analysis revealed the highest ER and PR positivity in the South West and the lowest in the South South region of the country, where TNBC and HER2+ tumours were relatively higher. This review provides an up-to-date synthesis of immunohistochemical subtype distribution of breast cancer in Nigeria, highlighting marked regional variability. The predominance of ER+ and luminal A subtypes supports the role of endocrine therapy in many patients, while high TNBC prevalence in certain regions underscores the need for improved access to chemotherapy and novel targeted treatments. These findings reinforce the importance of incorporating local epidemiological patterns into personalised breast cancer management strategies.

## Introduction and background

Breast cancer is an abnormal growth of tissue in the breast. It is the most common type of cancer affecting women worldwide, with 2.3 million cases recorded in 2022 [1]. In addition, breast cancer also represents the highest cancer mortality rates in women across the globe, with about 626,600 deaths due to the disease [2], and more women with lost disability-adjusted life years (DALY) due to breast cancer than any other type of cancer [1]. 

In Nigeria, 28,380 new cases of breast cancer were recorded in 2020, accounting for 22.7% of all new cases of cancer in the country [3]. Previous studies have revealed an increased breast cancer burden in Nigeria over the past decade [4], and the incidence is expected to increase further due to the westernisation of lifestyles, including delayed pregnancies, reduced breastfeeding, low age at menarche, lack of physical activity, low fibre diets, and better cancer registration and detection [5]. 

Female sex is the most important risk factor associated with breast cancer. This is linked to the sensitivity of breast tissue to circulating levels of oestrogen and androgens during the different phases of the reproductive cycle [6]. Other risk factors related to the prolonged exposure of the breast to estrogen include: early menarche, late menopause, prolonged use of oral contraceptives or oestrogen hormone replacement therapy, nulliparity, late age of first child birth, and non-breastfeeding [7]. Breast cancer also occurs in men and accounts for about 0.5% of all cases [1]. Breast cancers exhibit clinical, histologic, and biological heterogeneity [8]. In view of this, many studies have explored the histological and immunohistochemical patterns of breast cancer to group these tumours into classes for better understanding and clinical management. 

Classification of breast cancer into immunohistochemical subtypes is based on certain immunohistochemical markers such as estrogen receptor (ER), progesterone receptor (PR), human epidermal growth factor receptor 2 (HER2/neu) expression profile, and cell proliferation regulator (Ki-67). This molecular classification helps to predict the potential response to different treatment modalities as well as the prognosis of the cancer [9]. Four important immunohistochemical subtypes described include: luminal A, luminal B, HER2-positive (HER2+), and triple-negative breast cancer (TNBC).

Luminal A and luminal B possess ER and PR positivity. Luminal A subtypes are low-grade tumours with low proliferative and HER2/neu negative, and as such, patients with luminal A subtype tumours benefit from endocrine therapies, either with selective estrogen receptor modulators (tamoxifen) or with aromatase inhibitors (anastrozole) [10]. Luminal B subtypes may or may not have HER2/neu positivity and have a higher proliferative index. Luminal B subtypes make up 20% of invasive breast cancer cases and have a worse prognosis than luminal A. HER2+ possesses HER2 protein expression but lacks ER and PR, while TNBC subtypes lack ER, PR, and HER2 receptors. These two subtypes have a poorer prognosis and are only responsive to chemotherapy [11]. 

Knowledge of the molecular and immunohistochemical subtypes of breast cancer is vital for breast cancer management plans and prevention strategies. In many countries, testing for receptors and molecular subtypes is now a core part of the routine workup for breast cancer cases [9]. This promotes the ability to further individualise therapy to maximise therapeutic benefit. However, immunohistochemical receptors of breast cancer can vary from region to region; as such, there is a need for independent studies in every country to understand the prevalent subtype among various ethnic groups as a means to develop personalised patient management. This study is designed to highlight the various studies that have been carried out to review the immunohistochemical subtypes of breast cancer in Nigeria.

This paper was previously presented as a poster at the African Organisation for Research and Training in Cancer (AORTIC) Conference in Dakar, Senegal, on November 5, 2023.

## Review

Methods

Reporting Standards

This systematic review was carried out in strict adherence to the Preferred Reporting Items for Systematic Reviews and Meta-Analysis (PRISMA) guidelines [12]. The review was registered with the International Prospective Register of Systematic Reviews (PROSPERO) (registration ID: CRD420251128048).

Research Question

The research questions this study sought to answer were: (i) What is the prevalence of the different hormone receptor-positive breast cancers in Nigeria? (ii) What is the pattern of distribution of the immunohistochemical subtypes by region in Nigeria?

Eligibility Criteria

Studies were included if they met the following criteria: (i) conducted in Nigeria among Nigerian women, (ii) reported hormone receptor status (ER, PR, or HER2/neu) or immunohistochemical subtypes of breast cancer, and (iii) were primary research studies employing cross-sectional, retrospective, or prospective designs. Studies were excluded if they were conducted outside Nigeria, were review articles, commentaries, editorials, case reports, case series, or lacked relevant immunohistochemical data.

Information Sources And Search Strategy

A literature search was carried out for publications on the histological and immunohistochemical subtypes of breast cancer in Nigeria, in the PUBMED, Google Scholar, and African Journal Online (AJOL) databases from database inception to August 2025. The authors consulted with a librarian with experience in systematic searches to develop a search strategy. The search terms combined keywords and controlled vocabulary (Medical Subject Headings (MeSH) terms in PubMed) relating to breast cancer, histology, immunohistochemistry, and Nigeria. Boolean operators (“AND”, “OR”) were used to combine terms for comprehensive retrieval. The search strategy for the databases is summarised in Table 1. The results were then uploaded to Rayyan.ai (Cambridge, Massachusetts, United States), where duplicate studies were removed and additional screening was performed. 

**Table 1:** Search strategy

Database	Search Strategy	Hits
PubMed	("breast neoplasms"[MeSH Terms] OR ("breast"[All Fields] AND "neoplasms"[All Fields]) OR "breast neoplasms"[All Fields] OR ("breast"[All Fields] AND "cancer"[All Fields]) OR "breast cancer"[All Fields] OR ("breast neoplasms"[MeSH Terms] OR ("breast"[All Fields] AND "neoplasms"[All Fields]) OR "breast neoplasms"[All Fields] OR ("breast"[All Fields] AND "tumour"[All Fields]) OR "breast tumour"[All Fields]) OR "clinicopathology"[All Fields] OR ("anatomy and histology"[MeSH Subheading] OR ("anatomy"[All Fields] AND "histology"[All Fields]) OR "anatomy and histology"[All Fields] OR "histology"[All Fields] OR "histology"[MeSH Terms] OR "histologies"[All Fields]) OR ("immunohistochemistries"[All Fields] OR "immunohistochemistry"[MeSH Terms] OR "immunohistochemistry"[All Fields])) AND "nigeria*"[All Fields]	8,353
Google scholar	("breast cancer" OR "breast neoplasm" OR "breast tumour" OR "breast tumor") AND (immunohistochemistry OR histology OR clinicopathology) AND Nigeria	4,520
African Journal Online	(breast cancer OR breast neoplasms OR breast tumour OR breast tumor) AND (histology OR immunohistochemistry OR clinicopathology) AND Nigeria	114

Study Selection

Two reviewers independently screened titles and abstracts for relevance. Full-text articles of potentially eligible studies were retrieved and assessed against the inclusion criteria. Discrepancies were resolved through discussion, and a third reviewer was consulted if consensus could not be reached. The study selection process is summarised in a PRISMA flow diagram, detailing the number of studies identified, screened, excluded, and included.

Data Extraction

Data were extracted independently by two reviewers (COI and AD) using a standardised Excel spreadsheet (Microsoft Corporation, Redmond, Washington, United States). Extracted data included: author(s), year of publication, study design, sample size, study region, and detailed immunohistochemical and hormone receptor results. For studies reporting molecular subtypes, luminal A, luminal B, basal-like, HER2-enriched, and triple-negative classifications were recorded. Extracted data were cross-checked for accuracy.

Risk of Bias Assessment

Each included study was critically appraised for methodological quality using the JBI Critical Appraisal Tool for Analytical Cross-Sectional Studies [13]. This was carried out independently by two reviewers (MAM and MO). Assessment items included appropriateness of the sample frame, recruitment methods, measurement validity, data analysis, and completeness of outcome reporting. The results of this appraisal informed the interpretation of the findings and were used to assess the risk of bias across studies.

Data Synthesis

Data from included studies were summarised descriptively. Due to significant heterogeneity in the studies, a narrative synthesis was performed to describe the trend of distribution of receptors and molecular subtypes. The review also highlighted patterns and variations in prevalence across different Nigerian regions to contextualise findings.

RESULTS

**Figure 1:**
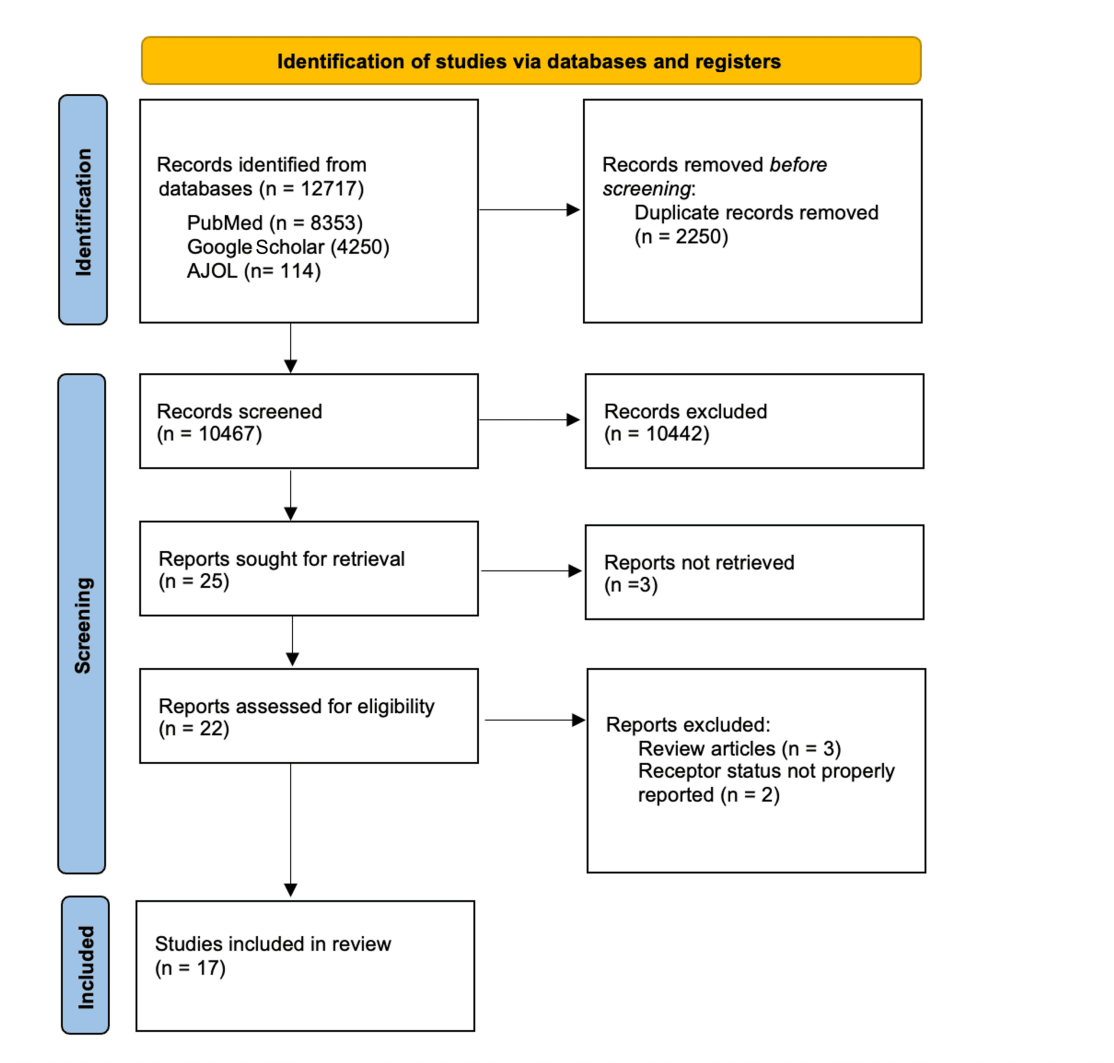
PRISMA flowchart for review PRISMA: Preferred Reporting Items for Systematic Reviews and Meta-Analysis; AJOL: African Journal Online

A total of 10,467 studies were screened after the initial database search and removal of duplicate studies. A total of 22 studies were assessed for eligibility, following which five were excluded, leaving 17 studies for the final review. This is summarised in Figure 1. 

Study Characteristics

Table 2 describes the characteristics of the studies included in the review. Most of these studies were carried out in tertiary centres, while a few were done at private facilities. The total number of participants in the 17 studies was 3017.

**Table 2:** Characteristics of included studies

Authors	Study type	Study Region in Nigeria	Year	Sample size, n
Tanimowo et al. [14]	Retrospective study	South-South	2019	61
Nwafor and Keshinro [15]	Retrospective study	South-West	2015	48
Ezike et al. [16]	Retrospective study	North-Central	2021	136
Eziagu et al. [17]	Retrospective study	South-South	2022	64
Omoruyi et al. [18]	Retrospective study	South-South	2018	147
Adebamowo et al. [19]	Prospective study	South West	2007	192
Adeniji et al. [20]	Prospective study	South-West	2020	251
Gukas et al. [21]	Retrospective study	North-Central	2005	36
Ukah et al. [22]	Prospective study	South-East	2017	123
Adeniji et al. [23]	Prospective study	North-Central	2016	171
Omoniyi-Esan et al. [24]	Prospective study	South-West	2015	136
Aliyu and Musa [25]	Prospective study	North-West	2020	259
Olasehinde et al. [26]	Retrospective study	South-West	2021	131
Wuraola et al. [27]	Retrospective study	South-West	2022	18
Tagar et al. [28]	Retrospective study	South-South	2024	41
Ayandipo et al. [29]	Retrospective study	South-West	2024	998
Adedokun et al. [30]	Retrospective study	South-West	2023	205

Out of the 17 studies meeting the inclusion criteria, 11 were retrospective in design [14-18,21,26-30] while six were prospective [19,20,22-25]. These studies were carried out between 2007 and 2024. Geographically, the studies were concentrated in Nigeria's South West region (n=8), followed by the South South (n=4), North Central (n=3), North West (n=1), and South East (n=1). Most of these studies were conducted in university teaching hospitals [14,17-29], with one in a private practice [15] and another in a district hospital [16].

Distribution of Hormone Receptor Status

A total of 16 studies (n = 2,846) reported the hormone receptor status of patients with breast cancer, providing data on ER, PR, and HER2/neu receptor status. This is described in Table 3. The prevalence of ER+ breast cancer ranged from 10.9% to 65.1%. The highest frequencies were observed in older or larger cohort studies such as those of Adebamowo et al. (65.1%) [19], Adedokun et al. (61.9%) [30], and Nwafor and Keshinro (54.2%) [15]. PR+ tumours demonstrated a similarly wide spectrum, with reported prevalence ranging from 12.5% to 70.7%. Several studies reported PR positivity rates below 30% [17,25,20], while others, such as that by Adebamowo et al. (54.7%) [19] and Nwafor and Keshinro (50.0%) [15], showed notably higher levels. HER2/neu positivity prevalence ranged from 5.2% to 62.5%. Most studies fell between 10-35% [15,20-22,26], though there were marked outliers such as that of Omoruyi et al. (8.2%) [24] at the lower end and Eziagu et al. (62.5%) [17] at the upper extreme. TNBC prevalence ranged from 12.2% to 79.3%. High TNBC frequencies (>50%) were specifically seen in studies such as that of Adeniji et al. (79.3%) [20], Tanimowo et al. (62.3%) [14], and Eziagu et al. (56.3%) [17], suggesting potential regional or methodological influences. 

**Table 3:** Distribution of hormone receptor status N/A: not available; ER+: estrogen receptor-positive; PR+: progesterone receptor-positive; HER2: human epidermal growth factor receptor 2; TNBC: triple-negative breast cancer

Authors, Year	Sample Size, n	ER+, n (%)	PR+, n (%)	HER2/neu+, n (%)	TNBC, n (%)
Tanimowo et al., 2019 [14]	61	11 (18.0%)	9 (14.8%)	20 (32.8%)	38 (62.3%)
Nwafor and Keshinro, 2015 [15]	48	26 (54.2%)	24 (50.0%)	6 (12.5%)	14 (29.2%)
Ezike et al., 2021 [16]	136	59 (43.4%)	53 (39.0%)	37 (27.2%)	53 (39.0%)
Eziagu et al., 2022 [17]	64	7 (10.9%)	8 (12.5%)	40 (62.5%)	36 (56.3%)
Omoruyi et al., 2018 [18]	147	N/A	N/A	12 (8.2%)	N/A
Adebamowo et al., 2007 [19]	192	125 (65.1%)	105 (54.7%)	10 (5.2%)	N/A
Adeniji et al., 2020 [20]	251	108 (43.0%)	70 (27.9%)	46 (18.3%)	199 (79.3%)
Gukas et al., 2005 [21]	36	9 (25.0%)	10 (27.8%)	9 (25.0%)	N/A
Ukah et al., 2017 [22]	123	52 (42.3%)	46 (37.4%)	14 (11.4%)	50 (40.7%)
Omoniyi-Esan et al., 2015 [24]	136	47 (34.6%)	34 (25.0%)	52 (38.2%)	45 (33.1%)
Aliyu and Musa, 2020 [25]	259	113 (43.6%)	42 (16.2%)	23 (8.9%)	N/A
Olasehinde et al., 2021 [26]	131	53 (40.5%)	42 (32.1%)	43 (32.8%)	57 (43.5%)
Wuraola et al., 2022 [27]	18	8 (44.4%)	N/A	6 (33.3%)	6 (33.3%)
Tagar et al., 2024 [28]	41	14 (34.1%)	11 (26.8%)	10 (24.4%)	17 (41.5%)
Ayandipo et al., 2024 [29]	998	469 (47.0%)	414 (41.5%)	180 (18.0%)	334 (33.5%)
Adedokun et al., 2023 [30]	205	127 (61.9%)	145 (70.7%)	70 (34.1%)	25 (12.2%)

Molecular Subtypes of Breast Cancer In Nigeria

Across the 10 studies that reported molecular subtypes (n = 2,220) [15-19,22-24,29,30], lumina A subtype had prevalence ranging from 4.7% to 61.9% Most studies recorded Luminal A prevalence between 33-52%, with particularly high rates recorded by Adebamowo (61.5%) [19], Adedokun (61.9%) [30], and Adeniji (50.3%) [23]. Luminal B tumours also showed considerable variation in their prevalence, with rates from 2.1% to 22.8%. Some studies reported luminal B prevalence well above 10% [15,16,24]. Basal-like subtype was reported in six of the 10 studies, with rates ranging from 12.5% to 40.7% [15,18,19,22-24]. Studies from the South East and South West regions [22,24] tended to report higher basal-like frequencies compared to those from other regions. This information is described in Table 4. 

**Table 4:** Distribution of molecular subtypes of breast cancer in Nigeria N/A: Not Available

Authors, Year	Sample Size, n	Luminal A, n (%)	Luminal B, n (%)	Basal-like, n (%)
Nwafor and Keshinro, 2015 [15]	48	19 (39.6%)	9 (18.8%)	14 (29.2%)
Ezike et al., 2021 [16]	136	46 (33.8%)	20 (14.7%)	N/A
Eziagu et al., 2022 [17]	64	3 (4.7%)	3 (4.7%)	N/A
Omoruyi et al., 2018 [18]	147	77 (52.4%)	19 (12.9%)	39 (26.5%)
Adebamowo et al., 2007 [19]	192	118 (61.5%)	4 (2.1%)	24 (12.5%)
Ukah et al., 2017 [22]	123	54 (43.9%)	6 (4.9%)	50 (40.7%)
Omoniyi-Esan et al., 2015 [24]	136	20 (14.7%)	21 (15.4%)	45 (33.1%)
Adeniji et al., 2016 [23]	171	86 (50.3%)	39 (22.8%)	43 (25.1%)
Ayandipo et al., 2024 [29]	998	364 (36.5%)	86 (8.6%)	N/A
Adedokun et al., 2023 [30]	205	127 (61.9%)	N/A	N/A

Regional Variation

In the South West region (eight studies; n = 1,929), ER positivity ranged from 34.6% to 65.1% [15,19,20,24,26,27,29,30]. PR positivity varied more widely, with rates from 25.0% to 70.7%. HER2+ tumours ranged from 5.2% to 38.2%. TNBC prevalence showed significant variation, from 12.2% to 79.3%. In the South South region (four studies; n = 313), ER positivity was markedly lower than in other regions, ranging from 10.9% to 34.1% [14,17,18,28]. PR positivity was also low, revealing a prevalence range of 8.9% to 26.8%. HER2 positivity showed more variations, with reported prevalence ranging from 8.2% to 62.5%. TNBC rates in this region were generally high, ranging from 41.5% to 62.3%. In the North Central region (three studies; n = 343), ER positivity ranged from 25.0% to 43.4% [16,21,23]. PR positivity ranged from 27.8% to 39.0%. HER2 positivity ranged from 25.0% to 27.2%. TNBC prevalence was reported in only one study at 39.0% [16]. In the South East region (one study; n = 123), ER positivity was 42.3%, PR positivity 37.4%, HER2 positivity 11.4%, and TNBC prevalence 40.7% [22]. In the North West region (one study; n = 259), ER positivity was 43.6%, PR positivity 16.2%, and HER2 positivity 8.9%; TNBC prevalence was not reported [25].

Risk of Bias Reporting

Assessment of study quality using the JBI's quality appraisal tool [13] indicated that most studies met the majority of the appraisal criteria. Specifically, 16 studies clearly defined inclusion criteria and measured outcomes using valid and reliable methods, with appropriate statistical analyses applied. Most studies also provided adequate information about the study subjects and setting. However, few studies identified potential confounding factors or described strategies to address them [26,27,30]. Two studies addressed confounding explicitly [26,27], while one study did not clearly define the sample [30]. Overall, all studies were deemed suitable for inclusion in the review. Table 5 summarises the appraisal outcomes for each study.

**Table 5:** Quality appraisal of included studies Y: Yes; N: No

Authors	Were the criteria for inclusion in the sample clearly defined?	Were the study subjects and the setting described in detail?	Was the exposure measured in a valid and reliable way?	Were objective, standard criteria used for measurement of the condition?	Were confounding factors identified?	Were strategies to deal with confounding factors stated?	Were the outcomes measured in a valid and reliable way?	Was appropriate statistical analysis used?	Overall appraisal
Tanimowo et al. [14]	Y	Y	N	Y	N	N	Y	Y	Include
Nwafor and Keshinro [15]	Y	Y	Y	Y	N	N	Y	Y	Include
Ezike et al. [16]	Y	N	Y	Y	N	N	Y	Y	Include
Eziagu et al. [17]	Y	Y	Y	Y	N	N	Y	Y	Include
Omoruyi et al. [18]	Y	N	Y	Y	N	N	Y	Y	Include
Adebamowo et al. [19]	Y	Y	Y	Y	N	N	Y	Y	Include
Adeniji et al. [20]	Y	Y	Y	Y	N	N	Y	Y	Include
Gukas et al. [21]	Y	N	Y	Y	N	N	Y	Y	Include
Ukah et al. [22]	Y	Y	Y	Y	N	N	Y	Y	Include
Adeniji et al. [23]	Y	N	Y	Y	N	N	Y	Y	Include
Omoniyi-Esan et al. [24]	Y	N	Y	Y	N	N	Y	Y	Include
Aliyu and Musa [25]	Y	N	Y	Y	N	N	Y	Y	Include
Olasehinde et al. [26]	Y	N	Y	Y	Y	Y	Y	Y	Include
Wuraola et al. [27]	N	N	Y	Y	Y	Y	Y	Y	Include
Tagar et al. [28]	Y	Y	Y	Y	N	N	Y	Y	Include
Ayandipo et al. [29]	Y	N	Y	Y	N	N	Y	Y	Include
Adedokun et al. [30]	Y	N	Y	Y	Y	Y	Y	Y	Include

Discussion

This systematic review synthesised data from 17 studies involving over 3,000 Nigerian patients with breast cancer, providing a comprehensive up-to-date overview of the immunohistochemical and molecular subtype distributions of breast cancer in the country. 

Global Comparisons

The distribution of breast cancer subtypes in Nigeria exhibits substantial variability and differs from global patterns. In our review, luminal A ranged from 4.7% to 61.9%, luminal B from 2.1% to 22.8%, and basal-like from 12.5% to 40.7%. Hormone receptor positivity also varied considerably: ER+ 10.9-65.1%, PR+ 8.9-70.7%, HER2+ 5.2-62.5%, and TNBC 12.2-79.3%.

By comparison, globally, luminal A accounts for approximately 50-60% of cases, luminal B 15-20%, HER2-enriched 10-15%, and TNBC approximately 20% [31,32]. Thus, while the upper end of Nigerian luminal A prevalence closely aligns with global estimates, the lower end is substantially lower, and TNBC is considerably more prevalent than in Western populations. For example, some Nigerian cohorts report TNBC rates up to 79.3%, compared with 15-20% globally [32].

Regional comparisons within sub-Saharan Africa provide more context. West African countries, including Nigeria, consistently report higher TNBC rates (up to 46%) than East African populations (21-27%) [33]. Similarly, ER and PR positivity tend to be lower in West Africa than in Europe or North America [34].

Several factors may explain these disparities. Biologically, shared West African ancestry has been associated with more aggressive tumour phenotypes, contributing to higher TNBC and lower hormone receptor expression, a pattern also observed among African American women [34]. Variability in pathology infrastructure, including inconsistent fixation, antibody selection, and interpretation criteria, may also lead to underestimation of ER and PR positivity, while evolving HER2 testing guidelines can impact reported rates [33]. 

Regional Variation and Implications

Regional disparities within Nigeria also warrant closer attention. Women from the South West exhibited a higher likelihood of presenting with ER+/PR+ disease, which has significant therapeutic implications, as these individuals are likely to benefit from endocrine treatments such as tamoxifen or aromatase inhibitors [19]. In contrast, cohorts from the South South displayed notably low hormone receptor expression [14,17,18,28], alongside disproportionately elevated HER2 positivity and TNBC rates exceeding 40% in certain studies [14,17,28]. These observations are particularly alarming, given that both HER2-enriched and triple-negative subtypes are associated with poorer prognoses and necessitate more resource-demanding treatment strategies, including trastuzumab or chemotherapy [35]. Data from North Central [16,21,23] and South East [22] regions revealed intermediate trends, with TNBC prevalence consistently around 40%, while a solitary study from the North West [25] indicated maintained ER expression but unusually low PR positivity, prompting inquiries into potential methodological or population-specific influences. This geographic variability underscores the diversity of breast cancer biology throughout Nigeria, which may be shaped by ethnic diversity, environmental factors, and differences in diagnostic methodologies.

Pathology Considerations

From a systems perspective, these findings underscore the critical need for investment in diagnostic infrastructure. Immunohistochemistry testing in Nigeria is not consistently accessible, and there is variability among laboratories regarding the quality of fixation, the antibodies employed, and the criteria for interpretation, all of which can affect the reported receptor status [19]. For instance, research has indicated that dependence on archival tissue or higher-grade tumors can lead to a significant underestimation of ER positivity and PR positivity [36]. Likewise, the evolving American Society for Clinical Oncology/College of American Pathologists (ASCO/CAP) guidelines for HER2 interpretation have had an impact on positivity rates globally [33]. It is imperative to achieve national standardization of immunohistochemical protocols, along with providing training for pathologists and oncologists, to ensure the accuracy and comparability of results. Additionally, enhancing cancer registries to incorporate molecular subtype data would assist in guiding resource allocation and monitoring trends over time.

Clinical and Public Health Implications

The clinical and public health implications of these findings are significant. The dominance of ER+ and luminal A cancers supports the need for the expansion of endocrine therapy initiatives throughout Nigeria. While tamoxifen is relatively inexpensive and widely accessible, there is a pressing requirement for enhanced efforts to promote adherence and to incorporate aromatase inhibitors for postmenopausal women [37]. Conversely, the significant burden of TNBC presents a more formidable challenge. In the absence of targeted hormonal or HER2 therapies, chemotherapy continues to serve as the primary treatment modality [38]. However, access to chemotherapy, and by extension radiotherapy, in Nigeria is inconsistent, often constrained by financial barriers, drug shortages, and treatment disruptions due to reliance on out-of-pocket expenses [26]. Likewise, the prevalence of HER2 positivity in specific areas highlights the critical need to enhance access to trastuzumab and other HER2-targeted therapies, which remain prohibitively costly for the majority of patients in Nigeria and Sub-Saharan Africa [39].

Limitations

There is considerable heterogeneity in study designs, sample sizes, and laboratory methodologies that limits generalizability. The absence of statistical testing for regional differences necessitates cautious interpretation, and incomplete reporting of molecular subtypes and confounders may affect observed ranges.

## Conclusions

This review emphasises that breast cancer in Nigeria is marked by a predominance of ER+ and luminal A subtypes, yet it also reveals a disproportionately high incidence of TNBC, particularly in specific areas. These results underscore the pressing necessity for region-specific cancer care strategies, guaranteeing universal access to affordable endocrine therapy, enhancing the availability of chemotherapy, and prioritising fair access to HER2-targeted and innovative TNBC treatments. Tackling diagnostic deficiencies and customising treatment protocols to align with Nigeria’s epidemiological context will be essential measures for improving outcomes for women affected by breast cancer.

Interpretation should be cautious due to heterogeneity in study design, immunohistochemical methods, and regional coverage, as well as limited statistical confirmation of regional differences. Nonetheless, this review underscores the importance of strengthening diagnostic capacity, standardising immunohistochemical protocols, and tailoring treatment strategies to Nigeria’s epidemiological context to improve outcomes for women with breast cancer.
